# The myth that slow test-takers are worse students: Implications for time-limited testing

**DOI:** 10.1111/medu.70215

**Published:** 2026-03-30

**Authors:** Saul J. Weiner, Yoon Soo Park, Morton Ann Gernsbacher, Kristina H. Petersen

**Affiliations:** 1 VA Center of Innovation for Complex Chronic Healthcare, Jesse Brown VA Medical Center, Chicago, Illinois, USA; 2 Department of Medicine, College of Medicine, University of Illinois at Chicago, Chicago, Illinois, USA; 3 Department of Medical Education, College of Medicine, University of Illinois at Chicago, Chicago, Illinois, USA; 4 Department of Psychology, University of Wisconsin-Madison, Madison, Wisconsin, USA; 5 Washington University School of Medicine, St. Louis, Missouri, USA

## Abstract

**Problem:**

Time-limited testing, a form of assessment in which participants have a fixed amount of time to complete an exam, remains a global standard across the medical education continuum from admissions through licensure and board certification. A wide-ranging literature, however, documents how speededness, the extent to which a test’s time limit alters individual performance, poses a threat to both validity and equity. Specifically, some students require more time than allotted to complete an exam. When afforded more time, they perform as well as or better than those who complete it within the time constraints. While some students who need more time seek and obtain accommodations, many others—because of stigma or a lack of awareness that they have a disability—fall outside this group. Among those who seek accommodation, some are denied despite a diagnosed disability because they lack childhood documentation.

**Myth:**

There is a lack of sufficient research evidence that people who complete academic tests rapidly are better at making appropriate clinical decisions or acting under time pressure, such as when a patient is bleeding into a surgical field. Rather, studies show experts know when to slow down to better manage a complex situation.

**Implications:**

Eliminating speededness on exams could be accomplished through power tests that functionally eliminate time pressure for virtually all test takers. At a practical level, this could be achieved by giving all examinees maximum accommodation, which is typically double time. Leveraging new technology and optimizing test design, including utilizing computerized adaptive testing, shortening stems, providing fewer response items and allowing remote proctoring, could partially off-set the added time and associated testing centre costs. Given the critical need to accurately and equitably assess performance on these gatekeeping exams, we recommend eliminating time limits expeditiously.

## 1 | BACKGROUND

High stakes examinations for entry into and advancement in the medical profession employ time-limited standardized testing formats across the assessment continuum, including for admission, licensure and specialty certification. In the United States and Canada, these include the Medical College Admission Test (MCAT) for admission, the United States Medical Licensing Examination (USMLE) and Medical Council of Canada Qualifying Examination (MCCQE) for licensure, and American Board of Medical Specialties (ABMS) certification examinations. Comparable time-limited examinations are used in the United Kingdom, Australia, New Zealand and Singapore.

Extra time is granted as an accommodation for qualifying disabilities when approved by the examining body. However, data on the number of examinees who request or receive extra time are not generally publicly disclosed. In the absence of such data, competing perceptions persist regarding the appropriateness of these accommodations.

These concerns, however, obscure a more fundamental one: Why are time limits imposed in the first place? Studies extending back over a hundred years have consistently shown time limits pose a threat to validity, the extent to which a test assesses what it purports to assess.^1–4^ They do so by preventing some examinees from fully considering all items, an effect known as “speededness.”

On time-limited exams in medicine, maximum accommodation is typically double-time, although 25% or 50% additional time appears to be more common.^5^ However, many who require additional time likely do not apply for it and, at least in the US – based on a survey of medical schools providing data not otherwise publicly available, many are denied accommodation.^6^ When this occurs appeals can become contentious, with candidates sometimes paying substantial sums for additional neuropsychological testing and legal representation.^6,7^ Concerns about fairness are reasonably evoked, as students without a disability—including those for whom English is a second language—are not eligible for the extra time.

Such dynamics, however, beg the question: Why all the fuss? Given a lack of evidence for timed testing and, in fact, substantial counterevidence, why not just give everyone the time they need to show what they know? Eliminating speededness requires, conceptually, giving every student as much time as they need to complete an exam, an approach known as untimed power testing.

We explore, here, the myth that time-limited testing measures something meaningful. Our critique of time-limited testing is grounded in Messick’s unified validity framework, which conceptualizes validity as integrated sources of evidence encompassing construct representation, sources of construct-irrelevant variance and the social consequences of score use.^8^ Within this framework, speededness represents a systematic source of construct-irrelevant variance targeting response process and consequential validity, thereby undermining equity, without enhancing inference about clinical competence.

We draw on research-based evidence from the fields of psychometrics, educational technology and cognition that support shifting current practices to allow all examinees the time necessary to complete assessments. We then discuss viable strategies for implementation. A move toward untimed power testing, employing advances in educational technology (computerized adaptive testing) and approaches from psychometric sciences (item response theory), can significantly improve assessment rigour and fairness. Developing and administering untimed examinations is no longer a question of possibility, but of a commitment to excellence in assessment and equity.

## 2 | THE MYTH

The myth is that if you know your stuff and are smart you should not need extra time.^9^ One report often cited in support of time-limited testing is a 2015 study, in which researchers in the United States examined the relationship between extra time on the MCAT and future academic performance.^10^ Comparing the group of matriculants who took the MCAT under standard conditions (n = 76 262) to those who received extra time (n = 449), the authors reported that the latter passed the USMLE Step examinations at lower rates than the former. They were also less likely to graduate from medical school. Such findings could suggest that needing extra time predicts lower academic performance.

However, there was a major limitation to the study that should raise questions about drawing such a conclusion: Specifically, the investigators did not have data on whether students who received accommodation on the MCAT also received accommodation on their USMLE exams. And, with a loss of accommodation, their performance would predictably drop, resulting in some cases in exam failure. As passing the USMLE exam is a nearly universal requirement at medical schools, higher exam failure also predicts lower graduation rates.

Unfortunately, the NBME, which administers the USMLE’s examinations, does not disclose its accommodations denial rate. Recently, they posted “accommodation requests approved fully or partially,” on their website which includes an undisclosed percentage of students granted extra-breaks *instead* of extra exam time—an outcome we have frequently observed and that we consider a denial.^11^ A national survey of student affairs officers led by one of us (KP) at medical schools across the nation supports a high denial rate.^6^ Of the students who received accommodations at their medical school who applied for USMLE accommodations, over half were denied. Within this group of students who were denied, over a third failed the exam after taking it unaccommodated. These findings comport with our experience serving on student promotion committees where we have repeatedly seen students denied accommodations despite receiving accommodations at the medical school they attend.

## 3 | CHALLENGING THE MYTH

A myth is a widely held but false belief or idea. Time-limited testing persists despite evidence going back to at least to 1921, including numerous studies demonstrating that student’s pace on a timed exam does not reflect performance.^12,13^ Any teacher administering tests to a relatively large class can conduct a simple experiment that challenges the stereotype that slow test takers are less capable: Compare students’ grades on exams with the order in which they complete them. As has been demonstrated repeatedly, the slow students do, on average, just as well as the fast ones.^12,14,15^ The experiment’s results, however, rely upon giving all the students the time they need to complete the exam. Ringing the bell before slower students have finished makes them *appear* less capable than faster students.

Indeed, there is growing evidence that the reverse is true when test items are challenging: students who take more time on difficult questions may perform better. In a landmark study published in *Nature Communications* in 2023, the authors asked, “Do intelligent people think faster?” and explored the question both through cognitive tests and functional brain imaging.^16^ Imaging demonstrated that slower but more accurate problems solvers exhibit higher functional connectivity. Knowing when to slow down because a problem is complex appears to be predictive of better performance.

Similar effects are observed in studies of one of the most cognitive of tasks, chess. Among expert players, the best is not necessarily the fastest. In fact, even in speed chess, stronger players spend more time on selective moves—the most challenging ones, reflecting their capacity to recognize complexity, resulting in fewer blunders.^17,18^

In studies of health professionals, similar findings emerge. For instance, in an analysis of 63 laparoscopic procedures performed by proficient novices (n = 43) and experts (n = 20), time pressure led to more errors (reduced suture precision), and increased mean force, affecting both groups, novices more so than experts. The authors concluded that mastery only partially compensates for the deleterious effects of time pressure.^19^ In another study, this one on diagnostic accuracy, senior residents were randomized to assign correct diagnoses to clinical cases with versus without time pressure.^20^ Both groups were afforded the same amount of time, but those in the experimental group received visual cues to give the impression that they were behind schedule. Physicians experiencing perceived time pressure made on average 37% more errors than control participants. Finally, in a different study of summative evaluation of medical students using multiple choice test questions, employing a data set with measures of both test-taking accuracy and speed, there was little relationship between them.^21^

Of note we found one study showing a correlation between accuracy of diagnosis and faster speed^22^: 75 residents were provided with a hypothetical scenario in which they are alone in an emergency department and have to see 25 patients in 30 min; the format was a written simulation consisting of cases of varying difficulty that they had to read and respond to. Under intense speededness, it is not surprising that the faster examinees were more accurate. Under such implausible or, at least rare, conditions one might prefer such physicians. However, the study did not assess how the slower and less accurate residents would have performed if speededness was removed. Multiple studies, as described above, would suggest they would have done as well and perhaps better on the more difficult cases.

Intuitively, the lack of correlation between speed and accuracy is not surprising. Each of us has our own pace when completing tasks, and many of us are especially careful when we recognize complexity and understand that the stakes are high. Would you rather have a physician who takes their time to diagnose your clinical signs and symptoms correctly, or one who is faster but less thorough?

That said, it is important to acknowledge that there are clinical tasks that are, by definition, time-limited, such as responding appropriately when a patient is in cardiac arrest or bleeding heavily into the surgical field. In some clinical contexts, time constraints may be part of the construct for conducting a medical history or physical examination. Hence, practical tests designed to assess through simulation performance under conditions where time matters should remain time-limited.

However, written assessments, including multiple choice tests, are not intended to assess speed in the workplace. Rather, they are designed to assess medical knowledge, problem solving and clinical reasoning. Numerous studies demonstrated that speededness in one task rarely transfers to other tasks, even when they are quite similar.^23^ For instance, in one classic experiment, subjects trained to rapidly press one set of keys on a keyboard were no better than a control group when given a new set of digits, letters and symbols.^24^ This distinction between multiple choice test-taking speed versus clinical skills in time-sensitive situations must be appreciated so that those who become physicians are not just the most efficient multiple-choice test takers in the country. Rather, we want physicians who are self-directed learners, compassionate, responsive to urgent needs, resilient, able to perform under intense pressure, and know when to pause to best address complexities.

## 4 | WHY THE MYTH IS HARMFUL

In a 2020 review, “Four Empirically Based Reasons Not to Administer Time-Limited Tests,” one of us (MAG) described three shortcomings of time-limited tests in addition to their inferior validity.^12^ One that should especially concern the medical profession is that time-limited tests are less equitable, because speededness impacts some groups more than others, such that construct validity is disproportionately diminished.^12^ As noted, examiners strive to mitigate inequities through the process of reviewing and granting, on a case-by-case basis, requests for accommodations on their exams. However, at least in the United States, military veterans,^25^ immigrants,^26^ first generation students, and low and even middle-income students with disabilities are less likely to seek accommodations either because of stigma or cost.^27^ And students with anxiety, depression and chronic health conditions often forgo accommodations because of stigma and/or fear of future career repercussions.^28–30^

Conversely, students from affluent families and those who attend private selective colleges are more likely to apply for and receive extended-time accommodations, at least in the US.^12^ Their parents more often have the education and resources to advocate for early intervention, diagnosis and treatment. These parents are more likely to be vigilant in keeping documentation, aware their child may need it at a later point in their educational journey. Fairness models in educational measurement underscore that adaptations to standardized procedures such as individualized accommodations to correct for speededness introduce additional inequities, reinforcing rather than mitigating validity threats.^31^ For these reasons, transitioning from time-limited to power testing should advance the equity missions of the organizations that administer tests.^32^

## 5 | FROM TIME-LIMITED TO FUNCTIONALLY UNTIMED POWER TESTING

In contrast to imposing strict time constraints, untimed power tests are intended to allow test takers to demonstrate knowledge and skills without feeling pressured by time limitations. The most straightforward approach to eliminate speededness for virtually all examinees is to give them all the time they need, which we define as the maximal accommodation available, typically double time for those who choose to use it.

One argument against such an adjustment is that it would increase cost at test centres. But is in-person proctoring the only option? During the pandemic, some US high stakes examinations, including the law school (LSAT) and graduate school (GRE) admission tests—but neither the MCAT nor USMLE—were administered remotely.^33^ In a study comparing performance across 14 097 US licensure exam candidates taking 11 exams in test centres or remotely, using live proctoring, where either option was available, there were no statistically significant differences, suggesting that cheating using current monitoring technology is uncommon.^34^ Similar studies of medical students taking the physician licensure exam (MCCQE) exam in Canada, and physicians taking a specialty certification exam in Europe, yielded similar findings.^35,36^ It would be useful to compare the costs as proctored home testing might facilitate power testing by mitigating perhaps the largest barrier to its implementation, namely increased cost of in person proctoring at testing centres. Regardless, given the extraordinarily high stakes implications of these examinations, is it not worth the extra expense to get the most valid results?

Another way, potentially, to mitigate the cost of lengthening the time allotted for examinations is to modify the exam itself: Shortening stems, reducing the number of multiple-choice response options (e.g., from five to three), and/or the total the number of response items shortens exam length.^37^ There is significant evidence that providing three answer options does not threaten validity nor reliability.^38^ Shorter stems reduce the amount of material an examinee must read, as does reducing the number of items. Notably, the effect of using any or all of these strategies on assessment depends on whether the content removed was essential to the validity of the resulting score interpretations.

Computerized adaptive testing (CAT) offers, perhaps, the most efficient strategy for shortening exam time and helping reduce speededness, although that was not its original intent. CAT was conceptualized over half a century ago in the 1970s as an innovation to improve measurement precision of test scores.^39^ Traditional approaches to measurement face limitations in assessment reliability; scores from very high- or very low-performing examinees are often unreliable due to measurement precision at further extremes of the score distribution. CAT was invented to resolve this measurement precision problem by allowing optimal measurement characteristics regardless of examinee score.^40^

The unintended benefit is that CAT allows customized assessments that are personalized to each learner’s response pattern, ultimately reducing the number of items to complete within a fixed period of time. The underlying psychometric theory behind CAT is that items are pre-calibrated (evaluated for their psychometric properties), thereby allowing an adaptive model to select the most optimal questions based on a candidate’s response pattern.^40^ A student with mastery of the material is quickly escalated to the most challenging questions and if they continue to select the correct answer, will complete the exam in relatively shorter time. Similarly, a student with a poor grasp of the material completes the exam in a short time after scoring incorrectly on the least challenging questions. Students at the centre of a distribution receive more questions to discriminate among small differences. It is not known, as far as we are aware, to what extent students in the middle group experience speededness. Giving maximal accommodation exam time to all test takers eliminates that concern.

Decades of research demonstrate that CAT generates assessments with greater validity evidence, including reliability and precision for making decisions.^41,42^ In contrast, traditional time-limited testing may be unreliable for learners at the ends of the score distribution.^43^ While not employed in medicine, CAT has become the standard testing platform in many high-stakes educational testing programs in the US, including the GRE (graduate schools), GMAT (business schools), NCLEX (nursing licensure), NREMT (paramedic) and more recently the SAT (college entrance).

## 6 | IMPLICATIONS

Switching from time-limited to functionally untimed power testing does come at a financial cost, especially if it involves adopting the technological and test design modifications proposed. In weighing the pros and cons, we draw on Van der Vleuten’s utility model for assessing professional competence. Besides reliability and validity, the model broadens criteria to include “the acceptability of the method to the stakeholders and the investment required in terms of resources.”^44^ The consequence of not making the change is the lost opportunity to administer assessments that are more valid and more equitable. Van der Vleuten’s utility model heavily weights these consequences in high-stakes medical assessments, in which any logistical advantages of time-limited testing are outweighed by losses in validity, educational impact and acceptability.^44^

A first step, as noted, would be to universalize maximal accommodation, immediately eliminating inequities related to who chooses to apply for and obtain accommodation, and hence resolving the consequent adverse effects on test validity. Doing so would also reduce the strain placed on students, medical schools and the disability accommodations staff who respond to requests for extra exam time, a process that is contentious and sometimes litigious.

The extended test times and consequent higher costs could be mitigated through a combination of strategies, foremost of which is the adoption of CAT technology. Additional strategies to consider include shortening question stems, reducing the number of words in reading passages and making questions more concise, all to the extent that the testing objectives can still be achieved. Potential cost savings through the wider implementation of remote proctoring warrants further study.

Further research on how best to offset universal accommodation, while needed, should not delay making the change. Based on the evidence, the question is not whether high stakes exams should remove time-limited testing; rather it is, why have they not? Why continue to administer tests under conditions that make them less valid, less reliable and less equitable?^12^ Gatekeepers into the profession of medicine have an opportunity to address a flawed assessment methodology that also likely contributes to significant inequities.
